# Surface-Guided Radiotherapy: Can We Move on from the Era of Three-Point Markers to the New Era of Thousands of Points?

**DOI:** 10.3390/bioengineering10101202

**Published:** 2023-10-16

**Authors:** Michalis Psarras, Despoina Stasinou, Theodoros Stroubinis, Maria Protopapa, Anna Zygogianni, Vassilis Kouloulias, Kalliopi Platoni

**Affiliations:** 1 Medical Physics Unit, 2nd Department of Radiology, Attikon University Hospital, Medical School, National and Kapodistrian University of Athens, 124 62 Athens, Greece; 2 Department of Radiation Oncology and Stereotactic Radiosurgery, Mediterraneo Hospital, 166 75 Athens, Greece; 3 Radiation Oncology Unit, 1st Department of Radiology, Aretaieion University Hospital, Medical School, National and Kapodistrian University of Athens, 115 28 Athens, Greece; 4 Radiation Oncology Unit, 2nd Department of Radiology, Attikon University Hospital, Medical School, National and Kapodistrian University of Athens, 124 62 Athens, Greece

**Keywords:** surface-guided radiotherapy, lasers/tattoos, patient positioning, setup time, dose reduction

## Abstract

The surface-guided radiotherapy (SGRT) technique improves patient positioning with submillimeter accuracy compared with the conventional positioning technique of lasers using three-point tattoos. SGRT provides solutions to considerations that arise from the conventional setup technique, such as variability in tattoo position and the psychological impact of the tattoos. Moreover, SGRT provides monitoring of intrafractional motion. Purpose: This literature review covers the basics of SGRT systems and examines whether SGRT can replace the traditional positioning technique. In addition, it investigates SGRT’s potential in reducing positioning times, factors affecting SGRT accuracy, the effectiveness of live monitoring, and the impact on patient dosage. Materials and Methods: This study focused on papers published from 2016 onward that compared SGRT with the traditional positioning technique and investigated factors affecting SGRT accuracy and effectiveness. Results/Conclusions: SGRT provides the same or better results regarding patient positioning. The implementation of SGRT can reduce overall treatment time. It is an effective technique for detecting intrafraction patient motion, improving treatment accuracy and precision, and creating a safe and comfortable environment for the patient during treatment.

## 1. Introduction

Radiotherapy, a cornerstone of cancer treatment, utilizes ionizing radiation to target cancer cells precisely while minimizing harm to healthy tissues. A critical aspect of radiotherapy is accurate patient positioning, which is pivotal in treatment planning and execution, as even minor deviations can compromise treatment effectiveness and increase the risk of side effects. Conventional methods include skin markings, tattoos, and laser alignment. The principle of laser/tattoo positioning is grounded in the notion that consistent patient setup within predetermined parameters translates to optimal treatment delivery, minimizing the risk of errors stemming from misalignment [1]. The advantages of conventional laser/tattoo positioning lie in its historical precedence and familiarity to clinicians. It offers a straightforward means of aligning patients with their treatment plans and facilitates reproducibility. However, this technique presents advantages and limitations that shape its utility for achieving optimal treatment outcomes [2].

The tattoos usually consist of 3–5-point markers, which may not be adequate for precise patient positioning in the translational and rotational axes. The patient’s skin mobility impacts the positions of markings and tattoos, which can lead to manual adjustments of the skin by radiation therapists (RTTs) to align the tattoos with the in-room lasers. This process can introduce uncertainty regarding patient positioning [2,3]. Furthermore, identifying tattoos from previous irradiation or other skin markings can be challenging [4,5]. These limitations underscore the need for more refined techniques that enhance precision while minimizing patient discomfort.

Surface-guided radiotherapy (SGRT) has emerged as an innovative solution to addressing these challenges and elevating treatment precision [6]. Leveraging advanced imaging technologies of non-ionizing radiation, SGRT has the potential to continuously monitor and guide patient positioning in real time in a non-invasive, patient-centered approach, enhancing treatment accuracy [7]. The core principle of SGRT involves comparing patients’ actual surface data with reference data obtained from the treatment plan using thousands of points to immediately detect deviations. 

The advantages of SGRT extend beyond accuracy. Many cancer patients receive tattoos as a permanent reminder of their difficult and stressful treatment period [8,9]. However, SGRT can eliminate the need for tattoos, as it is a positioning method that does not require them. Real-time patient monitoring mitigates intrafractional deviations, providing the ability to stop the treatment momentarily if necessary [10]. Furthermore, the continuous tracking of the patient’s surface enables the utilization of the SGRT system for the implementation of treatments with motion management techniques, such as deep inspiration breath hold (DIBH) [11,12]. Additionally, SGRT’s potential integration into adaptive radiotherapy strategies opens opportunities for personalized treatment approaches that account for anatomical variations over the treatment course [7,13].

This literature review aims to describe the fundamental principles of different SGRT systems. It investigates whether SGRT implementation can replace laser/tattoo positioning techniques for various treatment areas. Moreover, this review explores whether SGRT can decrease treatment times, identify the factors that may affect SGRT accuracy, assess the effectiveness of SGRT live monitoring, and evaluate whether SGRT can lower patient dosage.

### 1.1. SGRT Systems

#### 1.1.1. Catalyst^+^ HD (C-RAD, Upsala, Sweden)

C-RAD’s surface guidance system consists of the Catalyst^+^ HD system, comprising three ceiling-mounted units (two on the sides and one in the front, spaced 120° apart) installed in the treatment room. Each unit is equipped with a projector and a high-definition camera. Another system, the Sentinel, is installed in the computed tomography (CT) simulation room. The projectors emit visible structured light [14] at the blue wavelength (λ~405 nm) on the patient’s surface to create a 3D map of the surface topography, which the cameras capture in real time. This surface is then compared to the reference one generated by the Sentinel system, which uses laser scanning technology to reproduce the patient’s surface during CT simulation [14]. Comparison of the two surfaces is carried out using a non-rigid iterative closest point algorithm. The system calculates the patient’s surface displacements from the treatment isocenter in six degrees of freedom (lateral, longitudinal, vertical, yaw, roll, and pitch). Afterward, the user automatically performs all the shifts that must be made for the patients to be in the proper position. The system is equipped with an automated beam hold feature, which instantly stops the beam when the patient moves out of specific tolerance levels during the treatment. The three units are equipped with light-emitting diode (LED) projectors that display deviations in the optimal positioning on the patient’s surface. The colors red (λ~624 nm) and green (λ~528 nm) indicate mismatches between the patient’s surface and the reference.

#### 1.1.2. AlignRT Advance (VisionRT, London, UK)

AlignRT Advance is a surface imaging system that consists of three units attached to the ceiling of the treatment room. Two units are positioned laterally, while one is placed at the front, 90° apart. Each unit comprises a light projector and two high-definition cameras. The projectors emit structured light with a speckle pattern [14], which generates a 3D topography of the surface. The cameras use this topography to create a real-time 3D map of the patient’s surface. Using a rigid registration algorithm, this surface is compared to a reference generated by importing the skin contours from the CT simulation volumetric data. The reference surface represents the optimal treatment position relative to the prescribed treatment isocenter. The system indicates, via a monitor inside the treatment room, the shifts that must be applied in the three translational and three rotational axes for the two surfaces to agree. The registration of the real-time patient’s surface with the reference is carried out only within a specified area, so the user must define a region of interest (ROI) on the reference surface. The shape and position of the ROI depend on the treatment site. The tool “Postural Video” is a real-time visualization of the entire reference position outline via multiple angles through a monitor inside the treatment room. This tool supports the patient’s overall alignment relative to their reference position during setup and monitoring. Additionally, the system can automatically hold the beam when the patient position is out of the tolerance range during the treatment delivery. 

VisionRT provides an antidotally surface guidance system for patient positioning and monitoring in O-ring linear accelerators (linacs). This system is called AlignRT InBore, and it is an additional ring-shaped light projector/camera system mounted within the bore for intrafractional monitoring with six degrees of freedom. The positioning of the patient is performed by three units of projectors/cameras mounted on the treatment room ceiling, similar to the AlignRT Advance system, in the virtual isocenter according to the procedure above. When the patient is loaded into the treatment isocenter, monitoring is performed by AlignRT InBore with submillimeter precision, improving treatment accuracy. This also allows advanced treatment techniques such as DIBH to be performed on O-ring linacs [15].

#### 1.1.3. IDENTIFY (Varian Medical Systems, Palo Alto, CA, USA)

IDENTIFY is a surface image guidance system comprising of three ceiling-mounted units (two lateral and one frontal, 90° apart) in the treatment room. Each unit consists of a projector and two stereoscopic cameras. In addition, the system has two time of flight (ToF) cameras [16], one installed in the CT simulation room and the other in the treatment room. The ToF camera in the CT simulation room is used to acquire a head-to-toe 3D reference surface of the patient on the couch and the position of the immobilization devices in the simulation setup. This capture is utilized for the optimal placement and recognition of the immobilization devices and initial patient setup on the treatment couch. The verification is performed by the ToF camera, which is installed in the treatment room. The final patient positioning is performed using the three ceiling-mounted units. Each unit projects a speckle structured light to create the 3D topography of the surface, which the cameras use to reconstruct the 3D surface. Using a rigid algorithm, the patient’s surface is compared with the reference surface created by the skin outline from the CT simulation. Through a monitor in the treatment room, the system displays the translational and rotational offsets that must be performed for optimal patient positioning. The offset relative to the reference surface is based on the defined ROI. Also, the monitor in the treatment room visualizes the two surfaces: green, the real-time patient’s surface, and purple, the reference surface. When the two surfaces align within the tolerance levels, the green and purple surfaces fuse and become white. Areas of the surface that are not within the tolerance levels are visualized as red or blue. Red indicates that the surface is closer to the camera, and blue means that the surface is farther away. This way, additional information is provided along with the ROI registration. The newest version of IDENTIFY is also equipped with an automated beam-hold feature. 

#### 1.1.4. ExacTrac (Brainlab, Munich, Germany)

Another SGRT system is the ExacTrac Dynamic Surface from Brainlab (Munich, Germany). The system combines three different patient positioning and monitoring methods: optical surface imaging, thermal surface imaging, and X-ray imaging. It includes a single ceiling-mounted unit consisting of a structured light projector, two high-resolution cameras, and a thermal camera. The X-ray imaging system consists of two kV oblique X-ray tubes mounted on the floor next to the linac and two ceiling-mounted flat panel detectors. The initial positioning of the patient is performed using the SGRT system, and the final positioning is based on stereoscopic X-ray images of internal anatomy utilizing the integrated image-guidance radiotherapy (IGRT) system and updates surface tracking accordingly. The thermal camera provides a 2D thermal map of the patient’s surface. In combination with the optical surface imaging data, a hybrid 3D/thermal matrix is created, and an additional dimension is added. Consequentially, the system’s latency can be reduced during the monitoring of the intrafractional motion [17]. 

The latest update of the system incorporates a DIBH protocol, which involves integrating breath hold positioning with stereoscopic X-rays to verify internal anatomy. To implement this workflow, it is necessary to acquire free-breathing and DIBH planning CT datasets and contours. The free-breathing external outline is utilized with the SGRT system to position the patient. Subsequently, a point on the surface is set to generate a breathing trace that enables the monitoring of respiration and the patient’s surface relative to the DIBH external contour. Beam gating is executed using the surface camera information, and corrections are made using X-ray verification. Furthermore, the system furnishes visual feedback on the breath hold to the patient [18]. 

## 2. Materials and Methods

This literature review was conducted by searching the PubMed and Scopus platforms using keywords such as “Surface-Surface Guided Radiation Therapy”, “Surface Guided Radiotherapy”, “SGRT”, “tattoos”, and “patient positioning”. The focus was on papers published from 2016 onward to provide the latest information on SGRT systems. The search included papers comparing SGRT with laser/tattoo patient positioning techniques, recording mean or median values of residual errors, and reporting the root mean square (RMS) vector, as determined by IGRT patient positioning verification. Additionally, this review is based on papers that investigated the positioning times for SGRT and lasers/tattoos, factors affecting SGRT accuracy, the effectiveness of SGRT live monitoring, and SGRT’s potential to lower patient dosage.

## 3. Results

### 3.1. General

Table 1, Table 2, Table 3, Table 4 and Table 5 summarize the mean residual error values (in translational and rotational directions) of patients with cancer (CA) in the breast, head and neck (H and N), thorax, abdomen, and pelvis regions, respectively. The median values are presented in italics. Finally, the bold values in the tables indicate observations with statistically significant differences.

In the studies conducted by Walter et al. [19], Carl et al. [20], and Zhao et al. [21], the residual errors for SGRT were calculated theoretically, implementing the following workflow. All patients were positioned using the laser/tattoo technique. kVCBCT imaging was used to determine the translational errors of the laser/tattoo positioning (vLaser). At the same time, the SGRT system calculated the translational errors according to the actual surface compared to the reference (vSGRT). The translational errors in cases in which the patient was originally positioned by SGRT were calculated theoretically by the following formula: vSGRT-only = vLaser − vSGRT.

Results from combined anatomical regions, as in the studies by Stanley et al. [22] and Zhao et al. [21], not studied individually as those in our review were, were not included in this study. 

### 3.2. Breast

Table 1 displays the residual errors resulting from SGRT and tattoo positioning techniques in patients diagnosed with breast CA from eight studies published from 2017 to 2023. These studies provided mainly translational errors and the magnitude of the RMS vector. The rotational values were given in only two studies [23,24]. 

In general, the positioning of breast CA patients was improved by SGRT implementation. The residual errors were similar or statistically significantly reduced with SGRT. Moreover, the ranges of the RMS vector were from 2.4 mm to 6 mm and from 4.2 to 14 mm for SGRT and lasers/tattoos, respectively. In most studies, the RMS vector was statistically significantly reduced with SGRT. Kügele et al. [25] and Nguyen et al. [26] reported an increase in the percentage of treatment fractions that achieved their clinical criteria of translational errors using SGRT. Specifically, Kügele et al. [25] found that for tangential treatments, the percentages for which the clinical criteria (≤4 mm in translational errors) were fulfilled were 84% and 95% for lasers/tattoos and SGRT, respectively. The corresponding percentages for locoregional treatments were 54% and 70%, respectively. Nguyen et al. [26] calculated that the percentages of treatment sessions not exceeding ±2 mm in translational errors were 38% and 52% for lasers/tattoos and SGRT, respectively.

Hattel et al. [27] and Rigley et al. [28] provided the residual setup errors in the form of histograms, so apart from the RMS vector, the mean or median values could not be included in Table 1. Hattel et al. [27] found that for the longitudinal and rotational residual errors, there was no significant advantage of using SGRT compared to lasers/tattoos (*p* = 0.96 and *p* = 0.46, respectively). The lateral direction was statistically significantly better determined with SGRT (*p* < 0.001). On the other hand, the vertical direction was significantly better determined with lasers/tattoos than it was with SGRT (*p* < 0.001). It was not mentioned whether the difference in the RMS vector was statistically significant. 

### 3.3. Head and Neck/Brain

Table 2 displays the residual errors after utilizing SGRT and tattoo methods for positioning in patients with head and neck or brain tumors. It encompasses four studies from 2018 to 2023. The studies did not provide the RMS vector. 

The study by Flores-Martinez et al. [29] evaluated three patient groups. Group 1 utilized MV Cone Beam CT (MVCBCT) imaging to determine residual errors in patients with laser/tattoo positioning. Group 2 was positioned using SGRT, with MVCBCT performed to determine residual errors. Group 3 was positioned using the same technique, but residual errors were determined with kVCBCT imaging. This review only presents the comparison between Groups 1 and 2 using the MVCBCT modality to ensure unbiased results across different IGRT modalities.

Implementing SGRT for positioning in the H and N and brain regions caused a potential reduction in residual errors compared with laser/tattoo positioning. Carl et al. [20] observed statistically significant differences in translational errors between SRGT and laser/tattoo positioning in their study. The tattoo method exhibited better outcomes in the vertical and lateral directions, while SGRT demonstrated better results in the longitudinal direction. Wei et al. [30] found that the SGRT group showed a significant reduction in vertical translational errors for patients with oropharynx and oral cavity CA compared with the tattoo group (−1.7 mm for tattoos vs. −0.04 mm for SGRT, *p* = 0.01 and −2.8 mm for tattoos vs. −0.1 mm for SGRT, *p* < 0.01). In patients with oral cavity CA, SGRT also showed a marginally statistically significant reduction in longitudinal errors (1.5 mm for tattoos vs. 0.2 mm for SGRT, *p* = 0.08). Except for pitch errors in patients with oropharynx CA, rotational errors were comparable for SGRT and tattoos in each patient group (pitch errors: −0.08° for tattoos vs. 0.68° for SGRT group, *p* < 0.05). Also, they noticed that patients with oral cavity CA without SGRT presented significantly more fractions with vector shifts greater than 5 mm (50% for tattoos and 33% for SGRT). Flores-Martinez et al. [29] discovered a decrease in all the rotational errors, and the difference in pitch was statistically significant (*p* < 001). Chen et al. [31] found a statistically significant reduction in the median residual errors using the SGRT technique for patient positioning. The longitudinal errors were the only median value with a non-significant difference between SGRT and tattoos. They noticed that in the SGRT group, translational errors within 1 mm were observed in 88% of fractions, and rotational errors within 0.5° were observed in 84% of fractions in all treatment fractions. As for the tattoo group, translational errors within 1 mm were observed in 54% of fractions and rotational errors within 0.5° were observed in 38% of fractions.

### 3.4. Thorax

Table 3 presents the residual errors from comparing SGRT and laser/tattoo positioning techniques in patients with CA in the thorax region. This review examined five studies conducted between 2016 and 2023. 

In their studies, Zhao et al. [21], Carl et al. [20], and Walter et al. [19] found that the SGRT and laser/tattoo positioning techniques yielded similar outcomes in the thorax region. There were no significant differences observed in most cases. In a study conducted by Blace et al. [32], both techniques resulted in similar residual rotational and translation errors. However, SGRT decreased the percentage of treatment sessions with residual translation errors of over 5 mm when using SGRT (15% compared to 19% when using tattoos for positioning). In Qubala et al. [33], SGRT significantly increased the errors in thoracic CA patients’ vertical, longitudinal, and RMS vectors from 0.2 ± 2.3 mm to 1.9 ± 2.8 mm, from 0.1 ± 3 mm to −1.1 ± 2.9 mm, and from 5 ± 1.6 mm to 5.7 ± 2.2 mm, respectively. The rotational errors were lower with SGRT for the thoracic cohort, particularly in pitch (*p* = 0.009).

### 3.5. Abdomen

Table 4 shows the residual errors obtained by comparing the SGRT and laser/tattoo positioning techniques in patients with CA in the abdominal region. This review focused on three studies carried out from 2016 to 2018. 

A study conducted by F. Walter et al. [19] found a trend toward increased residual errors with SGRT implementation. Carl et al. [20] observed a statistically significant reduction (*p* < 0.001) in the longitudinal and lateral directions using the laser/tattoo patient positioning technique in the abdominal region (Long: from 1.99 ± 5.25 to 1.56 ± 4.18 mm and Lat: from −0.46 ± 4.85 to −0.06 ± 4.03 mm). By contrast, Stanley et al. [22] found a statistically significant reduction (*p* < 0.05) in the RMS vector with SGRT from 10 ± 5 to 5 ± 3 mm.

### 3.6. Pelvis

Table 5 presents the residual errors of patients with pelvic region CA after positioning using SGRT and tattoo techniques. The table includes three studies conducted between 2016 and 2023. 

In the pelvic area, two out of three studies [16,22] found that SGRT yielded better outcomes than lasers/tattoos regarding patient positioning. In a study conducted by Walter et al. [19], no significant differences were observed, except for a statistically significant difference in the longitudinal direction in which tattoos were more effective than SGRT. According to Qubala et al. [33], SGRT resulted in a significant decrease in the RMS vector, from 7.1 ± 2.3 to 6.6 ± 2.3 mm, compared with tattoos. The study also found that SGRT led to lower rotational corrections, with a significant difference in pitch (*p* = 0.002). Mannerberg et al. [34] discovered that using SGRT for patient positioning significantly improved translational error values. The results showed a notable decrease in both lateral and vertical direction, with SGRT reducing the median values from 1.9 mm (range: 0–15.2 mm) to 1.1 mm (range:0–5.6 mm) (*p* = 0.02) and from 2.6 mm (range: 0–12.6 mm) to 2.2 mm (range: 0–9.3 mm) (*p* = 0.04), respectively. The median vector offset was 4.7 mm (range: 0–10.4 mm) for SGRT and 5.2 mm (range: 0.41–17.3 mm) for tattoos (*p* = 0.01). The probability of positioning a patient within a total vector offset of 7 mm was 84% for SGRT and 71% for lasers/tattoos.

**Table 1:** The residual errors resulting from SGRT and laser/tattoo positioning techniques in patients diagnosed with breast cancer.

Authors	Sample	SGRT Device	Mean Residual Errors ± SD or *Median Residual Errors (Range)*													
			Vertical (mm)		Longitudinal (mm)		Lateral (mm)		RMS (mm)		Yaw (°)		Pitch (°)		Roll (°)	
			SGRT	Tattoos	SGRT	Tattoos	SGRT	Tattoos	SGRT	Tattoos	SGRT	Tattoos	SGRT	Tattoos	SGRT	Tattoos
Stanley et al. (2017) [22]	600–900 frx (Both setup techniques were used for each patient)	Catalyst HD	N/A	N/A	N/A	N/A	N/A	N/A	**6 ± 2**	**14 ± 7**	N/A	N/A	N/A	N/A	N/A	N/A
Cravo Sa (2018) [23]	10 Pts SGRT vs. 10 Pts tattoos (breast FB)	AlignRT	−2.1 ± 1.4	3.1 ± 2.9	0.5 ± 2.9	−2.2 ± 3.3	**−0.2 ± 1.3**	**2.2 ± 2.9**	N/A	N/A	*−0.01*	*−0.28*	0.3 ± 1.22	0.25 ± 1.11	***0.72***	***0.05***
Hattel et al. (2019) [27]	99 frx SGRT vs. 44 frx tattoos	AlignRT	N/A	N/A	N/A	N/A	N/A	N/A	4.2	5.4	N/A	N/A	N/A	N/A	N/A	N/A
Kügele et al. (2019) [25]	37 Pts SGRT vs. 26 Pts tattoos (tangential treatment)	Catalyst (1 unit)	**1.5 ± 1.7**	**0.6 ± 3.7**	0.4 ± 1.5	0.8 ± 3.7	−0.5 ± 1.4	−0.6 ± 3.3	***2.4 (0–8.1)***	***4.2 (0–19.7)***	N/A	N/A	N/A	N/A	N/A	N/A
	42 Pts SGRT vs. 34 Pts tattoos (locoregional treatment)	Catalyst HD	**−0.3 ± 2.9**	**0.7 ± 3.1**	−0.1 ± 2.8	0.1 ± 3.3	**−0.5 ± 2.8**	**0.1 ± 3.4**	***4 (0–13.5)***	***4.7 (0–18.7)***	N/A	N/A	N/A	N/A	N/A	N/A
Rigley et al. (2020) [28]	191 frx SGRT vs. 197 frx tattoos (right breast FB)	AlignRT	N/A	N/A	N/A	N/A	N/A	N/A	**4.7**	**5.2**	N/A	N/A	N/A	N/A	N/A	N/A
	191 frx SGRT vs. 201 frx tattoos (left breast DIBH)		N/A	N/A	N/A	N/A	N/A	N/A	**4.5**	**7.6**	N/A	N/A	N/A	N/A	N/A	N/A
Nguyen et al. (2021) [26]	10 Pts SGRT vs. 10 Pts tattoos (breast FB)	AlignRT (Installed in O-ring linac)	**2 ± 2**	**3 ± 4**	**−1 ± 3**	**−1 ± 6**	**3 ± 2**	**0 ± 5**	N/A	N/A	N/A	N/A	N/A	N/A	N/A	N/A
Svestad et al. (2022) [35]	25 Right Breast Pts (Both setup techniques were used for each patient; half of the fractions with SGRT and the other half with tattoos)	AlignRT	**−3.2 ± 1.1**	**−0.4 ± 2.7**	0.2 ± 2.7	0.5 ± 3.5	0.7± 1.6	−0.1 ± 1.3	N/A	N/A	N/A	N/A	N/A	N/A	N/A	N/A
Kang et al. (2023) [24]	38 breast Pts (Both setup techniques were used for each patient; 3 frx with SGRT and 3 tattoos with SGRT)	AlignRT (installed in O-ring linac)	**1.9 ± 1.2**	**2.7 ± 1.6**	**2.9 ± 2.1**	**2 ± 1.2**	1.9 ± 0.7	2.1 ± 1	N/A	N/A	0.51 ± 0.26	0.51 ± 0.24	0.3 ± 0.22	0.32 ± 0.3	**0.19 ± 0.13**	**0.29 ± 0.22**

Abbreviations: frx = fractions, Pts = patients, FB = free-breathing, SGRT = surface-guided radiotherapy, N/A = not available. The median values are represented in italics. The bold values in the table indicate observations with statistically significant differences.

**Table 2:** The residual errors resulting from SGRT and tattoo positioning techniques in patients diagnosed with H and N/brain cancer.

Authors	Sample	SGRT Device	Mean Residual Errors ± SD or *Median Residual Errors (Range)*											
			Vertical (mm)		Longitudinal (mm)		Lateral (mm)		Yaw (°)		Pitch (°)		Roll (°)	
			SGRT	Tattoos	SGRT	Tattoos	SGRT	Tattoos	SGRT	Tattoos	SGRT	Tattoos	SGRT	Tattoos
Carl et al. (2018) [20]	689 frx (Patients were positioned using tattoos and verified with IGRT. Residual errors for SGRT were calculated theoretically)	Catalyst HD	**0.13 ± 2.88**	**−0.03 ± 2.27**	**0.17 ± 2.11**	**0.29 ± 1.89**	**−0.21 ± 2.14**	**−0.18 ± 1.71**	N/A	N/A	N/A	N/A	N/A	N/A
Wei et al. (2020) [30]	10 Pts SGRT vs. 10 Pts tattoos (oropharynx)	AlignRT	**0.38 ± 0.79**	**−1.68 ± 1.09**	0.03 ± 0.86	0.63 ± 1.52	0.36 ± 1.22	0.09 ± 0.95	−0.14 ± 0.40	−0.09 ± 0.34	**0.68 ± 0.49**	**−0.08 ± 0.95**	0.2 ± 1.21	0.26 ± 1.07
	10 Pts SGRT vs. 10 Pts Tattoo (oral cavity)		**−0.06 ± 1.16**	**−2.77 ± 1.33**	0.19 ± 0.89	1.5 ± 1.46	−0.49 ± 1.11	−0.11 ± 1.82	0.12 ± 0.44	0.10 ± 0.19	0.15 ± 0.42	0.1 ± 0.65	0.29 ± 0.81	−0.33 ± 0.90
	10 Pts SGRT vs. 10 Pts tattoos (nasopharynx/sinonasal)		−0.32 ± 0.58	−0.04 ± 1.40	−0.33 ± 0.54	0.39 ± 1.26	−0.25 ± 0.63	−0.38 ± 1.9	0.11 ± 0.31	0.03 ± 0.35	0.44 ± 0.66	0.18 ± 0.52	−0.45 ± 1.06	0.01 ± 0.93
Flores-Martinez (2020) [29]	107 frx SGRT vs. 117 frx tattoos	AlignRT (installed in O-ring linac)	N/A	N/A	N/A	N/A	N/A	N/A	0.79 ± 0.66	0.93 ± 0.98	**0.53 ± 0.45**	**1.24 ± 0.97**	0.54 ± 0.49	0.80 ± 0.76
Chen (2023) [31]	20 Pts SGRT vs. 20 Pts tattoos (SRS/SRT)	AlignRT	***0.4 (0.2–0.7)***	***1.1 (0.5–1.6)***	*0.7 (0.3–1.1)*	*1 (0.4–1.7)*	***0.3 (0.1–0.7)***	***0.9 (0.5–1.5)***	***0.2 (0.05–0.5)***	***0.75 (0.3–1.15)***	***0.4 (0.1–0.6)***	***0.6 (0.2–1.3)***	***0.2 (0.1–0.35)***	***0.6 (0.2–0.85)***

Abbreviations: frx = fractions, Pts = patients, SGRT = surface-guided radiotherapy, IGRT = image-guided radiotherapy, N/A = not available. The median values are represented in italics. The bold values in the table indicate observations with statistically significant differences.

**Table 3:** The residual errors resulting from SGRT and tattoo positioning techniques in patients diagnosed with thoracic cancer.

Authors	Sample	SGRT Device	Mean Residual Errors ± SD													
			Vertical (mm)		Longitudinal (mm)		Lateral (mm)		RMS (mm)		Yaw (°)		Pitch (°)		Roll (°)	
			SGRT	Tattoos	SGRT	Tattoos	SGRT	Tattoos	SGRT	Tattoos	SGRT	Tattoos	SGRT	Tattoos	SGRT	Tattoos
Walter et al. (2016) [19]	8 Pts—25 frx (Patients were positioned using tattoos and verified with IGRT. Residual errors for SGRT were calculated theoretically)	Catalyst (1 unit)	0.5 ± 3.2	0.6 ± 4.1	−5 ± 7.9	−2 ± 3.5	0.6 ± 2.6	0.7 ± 2.5	N/A	N/A	N/A	N/A	N/A	N/A	N/A	N/A
Carl et al. (2018) [20]	460 frx (Patients were positioned using tattoos and verified with IGRT. Residual errors for SGRT were calculated theoretically)	Catalyst HD	**−0.01 ± 4.64**	**0.22 ± 4.18**	**−1.14 ± 4.66**	**−0.65 ± 3.65**	**−0.13 ± 4.67**	**−0.21 ± 4.06**	N/A	N/A	N/A	N/A	N/A	N/A	N/A	N/A
Blake et al. (2022) [32]	19 Pts SGRT vs. 17 Pts tattoo	AlignRT	**2 ± 3**	**−1 ± 2**	0 ± 3	0 ± 5	0 ± 3	0 ± 3	N/A	N/A	0.3 ± 1.5	0.2 ± 1.4	0.3 ± 1.1	0.4 ± 1.2	**0 ± 1.3**	**−0.4 ± 1.3**
Zhao et al. (2022) [21]	25 Pts—202 frx (Patients were positioned using tattoos and verified with IGRT. Residual errors for SGRT were calculated theoretically)	AlignRT	3.1 ± 2.4	3.2 ± 3.1	4.6 ± 4.4	3.4 ± 3.9	2.6 ± 2.5	2.9 ± 2.8	N/A	N/A	N/A	N/A	N/A	N/A	N/A	N/A
Qubala (2023) [33]	10 Pts—100 frx (Both setup techniques were used for each patient)	AlignRT	**1.9 ± 2.8**	**0.2 ± 2.3**	**−1.1 ± 2.9**	**0.1 ± 3**	0.5 ± 1.4	0.4 ± 2.2	**5.7 ± 2.2**	**5 ± 1.6**	**−0.5 ± 0.5**	**−0.2 ± 0.8**	**0 ± 0.5**	**0.5 ± 0.8**	0.2 ± 0.5	0.3 ± 0.9

Abbreviations: frx = fractions, Pts = patients, SGRT = surface-guided radiotherapy, IGRT = image-guided radiotherapy, N/A = not available. The bold values in the table indicate observations with statistically significant differences.

**Table 4:** The residual errors resulting from SGRT and tattoo positioning techniques in patients diagnosed with abdominal cancer.

Authors	Sample	SGRT Device	Mean Residual Errors ± SD													
			Vertical (mm)		Longitudinal (mm)		Lateral (mm)		RMS (mm)		Yaw (°)		Pitch (°)		Roll (°)	
			SGRT	Tattoos	SGRT	Tattoos	SGRT	Tattoos	SGRT	Tattoos	SGRT	Tattoos	SGRT	Tattoos	SGRT	Tattoos
Walter et al. (2016) [19]	4 Pts—21 frx (Patients were positioned using tattoos and verified with IGRT. Residual errors for SGRT were calculated theoretically)	Catalyst (1 unit)	2.1 ± 5.5	2.1 ± 2.7	2.6 ± 1.8	−0.4 ± 1.2	0.3 ± 2.2	2.2 ± 1.3	N/A	N/A	N/A	N/A	N/A	N/A	N/A	N/A
Stanley et al. (2017) [22]	600–900 frx (setup from both techniques)	Catalyst HD	N/A	N/A	N/A	N/A	N/A	N/A	**5 ± 3**	**10 ± 5**	N/A	N/A	N/A	N/A	N/A	N/A
Carl et al. (2018) [20]	630 frx (Patients were positioned using tattoos and verified with IGRT. Residual errors for SGRT were calculated theoretically)	Catalyst HD	0.59 ± 5.58	0.68 ± 3.93	**1.99 ± 5.25**	**1.56 ± 4.18**	**−0.46 ± 4.85**	**−0.06 ± 4.03**	N/A	N/A	N/A	N/A	N/A	N/A	N/A	N/A

Abbreviations: frx = fractions, Pts = patients, SGRT = surface-guided radiotherapy, IGRT = image-guided radiotherapy, N/A = not available. The bold values in the table indicate observations with statistically significant differences.

**Table 5:** The residual errors resulting from SGRT and tattoo positioning techniques in patients diagnosed with pelvic cancer.

Authors	Sample	SGRT Device	Mean Residual Errors ± SD or *Median Residual Errors (Range)*													
			Vertical (mm)		Longitudinal (mm)		Lateral (mm)		RMS (mm)		Yaw (°)		Pitch (°)		Roll (°)	
			SGRT	Tattoos	SGRT	Tattoos	SGRT	Tattoos	SGRT	Tattoos	SGRT	Tattoos	SGRT	Tattoos	SGRT	Tattoos
Walter et al. (2016) [19]	13 Pts—108 frx (Patients were positioned using tattoos and verified with IGRT. Residual errors for SGRT were calculated theoretically)	Catalyst (1 unit)	1.6 ± 2.2	1 ± 1.1	**−1.7 ± 2.8**	**0.4 ± 1.4**	−0.9 ± 1.5	−0.9 ± 1.4	N/A	N/A	N/A	N/A	N/A	N/A	N/A	N/A
Mannerberg et al. (2021) [34]	20 Pts SGRT vs. 20 Pts tattoos	Catalyst (1 unit)	***2.2 (0–9.3)***	***2.6 (0–12.6)***	*1.8 (0–9.6)*	*1.6 (0–15.2)*	***1.1 (0–5.6)***	***1.9 (0–15.2)***	***4.7 (0–10.4)***	***5.2 (0.41–17.3)***	N/A	N/A	N/A	N/A	N/A	N/A
Qubala et al. (2023) [33]	11 Pts—100 frx (Both setup techniques were used for each patient)	AlignRT	2.4 ± 3.2	1.9 ± 3.4	−0.7 ± 2.4	0.1 ± 4	0.2 ± 3.1	1.4 ± 2.6	**6.6 ± 2.3**	**7.1 ± 2.3**	0 ± 0.5	0.2 ± 0.5	**0.1 ± 1.2**	**−0.6 ± 1.2**	−0.3 ± 0.7	−0.3 ± 0.4

Abbreviations: frx = fractions, Pts = patients, SGRT = surface-guided radiotherapy, IGRT = image-guided radiotherapy, N/A = not available. The median values are represented in italics. The bold values in the table indicate observations with statistically significant differences.

## 4. Discussion

### 4.1. Breast

Implementing SGRT generally improved the positioning of breast cancer patients. The residual errors were either significantly reduced or similar to those of SGRT. 

This was also confirmed by Kost et al. [36], who investigated the positioning accuracy in four groups of breast/chest wall CA patients (regional irradiation with and without DIBH and locoregional irradiation with and without DIBH). Table 1 does not include the above study, as only the setup errors for the skin, CW, and heart were used and not the translational and rotational errors. The setup error for each landmark was defined as the mean and maximum distance between the projected planning structure and the delineated structure on the portal image. They found a significant reduction (*p* < 0.001) in the skin setup errors with SGRT in all patient groups. In all patients (tattoos vs. SGRT), the mean errors for the skin decreased from 3.5 mm to 2.3 mm (*p* < 0.001). Setup errors for the CW were not significantly different from those for SGRT in regional groups but were significantly reduced (*p* = 0.04) in the group of patients receiving locoregional irradiation with DIBH using SGRT. In all patients, the mean errors for the CW were reduced from 3.1 mm to 3.0 mm (*p* = 0.21). No significant difference in setup errors was observed for the heart. 

According to the data presented in Table 1, the standard deviations (SDs) of the translational errors with SGRT were consistently smaller. Therefore, the reproducibility of patient positioning may be better with the SGRT technique. Two studies in Table 1 [23,35] also provided data on random errors (σ), which showed a similar trend. Svestad et al. [10] calculated the planning target volume (PTV) margins using the van Herk formula (2.5Σ + 0.7σ) [13]. According to their calculations, PTV margins were reduced from 8 to 4mm and from 10 to 8 mm in the vertical and longitudinal directions. The PTV margins remained unchanged in the lateral direction. Hence, SGRT implementation can potentially reduce PTV margins, thus leading to lower toxicity to the patient’s healthy tissue.

Nonetheless, two studies in Table 1 [27,35] reported statistically significant improvements in the vertical direction of residual errors using the conventional patient positioning technique, commenting on the “lower thorax” effect. Hattel et al. [27] suggested that patients were more relaxed during the treatment sessions compared with CT simulation. When the SGRT technique was implemented for patient positioning, a couch shift upward compensated for a lower thorax surface. When the IGRT modality was applied for verification using the spine as the primary match structure for image registration, it revealed a higher position of the vertebrae. Svestad et al. [35] observed that in most treatment fractions, patients had an increasingly lower thorax from the moment they were positioned until kVCBCT imaging. Therefore, this vertical displacement was compensated for with a couch shift vertically upward. They explained that the patients tended to relax more when treatment personnel left the treatment room. Both studies agreed that the three-point markers were less sensitive to the “lower thorax” effect.

The accuracy and reproducibility of a patient’s arm positioning are challenging in breast radiotherapy. Li et al. [37] showed that misalignment of the arm in an unsuitable position greatly impacted both the breasts and the lymph nodes, leading to deviations in the dose distributions to the PTV and organs at risk (OARs). Kapanen et al. [38] investigated the arm positioning of breast CA patients implementing the conventional setup technique. They found that in 65% and 56% of patients, an arm position correction was needed at least once during the treatment when using wrist-hold and rod-hold fixation devices, respectively. The implementation of the SGRT could provide a solution to these concerns by providing information on the arm and chin position. Dekker et al. [12] assessed the accuracy of the arm position, determining the clavicle position in 84 patients who received local or locoregional breast radiotherapy and were positioned utilizing SGRT. They found that 91.6% of the patients had a successful arm setup based on the clavicle rotation. The mean clavicle rotation was 0.4° ± 2°, ensuring the accuracy and precision of treatment delivery. 

Another parameter that can affect the accuracy of positioning in breast CA patients is the shape and size of the ROI. Laaksomaa et al. [39] estimated the positioning accuracy in patients with breast CA utilizing three different ROIs. The first was a breast-shaped ROI (B-ROI). The second was an ROI similar to the B-ROI but excluding a central O-shaped soft tissue region of the breast (O-ROI). The third was an ROI shaped as an upside-down T (T-ROI) consisting of the sternum and belt area under the breasts. The verification of the positioning was performed with kV imaging. Fewer variations were found when ROIs were utilized for the setup in comparison with lasers/tattoos. T-ROI provided the smallest rotational errors, as it represented the vertebral rotation most accurately. Sauer et al. [40] investigated the influence of the ROI’s size and shape in positioning. Various sizes of ipsilateral breast-shaped ROIs were compared. A 5 cm increase in the size of an ipsilateral breast-shaped ROI resulted in a statistically significant reduction of 25.4% in the rotational variation and a reduction of 8.9% in the translation variation, which did not reach statistical significance. In addition, twelve differently shaped ROIs were evaluated. The ROIs contained a combination of various anatomical regions, like the ipsilateral breast, the contralateral breast, the sternum, the axilla, a belt underneath the breasts, and the lymph region. A T-ROI shape very similar to that in a previous paper was included. The absolute deviations between SGRT-based shifts and clinically applied kVCBCT-based shifts were evaluated. An ROI that included the ipsilateral breast, sternum, and a belt underneath the breasts showed the best results in reducing overall translational and rotational deviation compared with kVCBCT-based residual errors. They investigated the potential correlation between SGRT-based shifts of the ROI above and breast volume. Negligible correlations were found between a breast size of <1400 cm^3^ and shift size. For a breast size of >1400 cm^3^, T-ROI had slightly better results. Both studies evaluated the impact of the ROI shape with the same SGRT device.

SGRT seems to reduce the positioning time in breast CA patients. Kang et al. [24] found that the average total setup time was significantly reduced by 5% owing to a reduction in imaging time by approximately 13%. Imaging time is defined from the beginning of the kVCBCT until the couch shift is applied. A decrease in imaging time can result from an increased initial setup accuracy. The same conclusion was drawn by Penninkhof et al. [41] because the patient posture and the arm and chin position could be adjusted a priori on the basis of surface guidance. They evaluated 47 DIBH breast CA patients positioned with SGRT compared with 25 DIBH breast CA patients positioned with lasers/tattoos. SGRT positioning was completed within 3.5 min for 95% of the fractions, whereas 85% were completed within 3.5 min with laser/tattoo positioning. Nguyen et al. [26] indicated that SGRT provided significant (*p* = 0.038) time sparing compared with tattoos, measuring treatment times of 10.9 ± 1.4 vs. 11.3 ± 2.1 min, respectively. Batin et al. [42] found a 45% shorter setup time for chest wall CA patients treated with proton irradiation using SGRT in comparison to a combination of laser/tattoos and orthogonal kV imaging. On the contrary, Kost et al. [36] found a statistically significant increase in the setup time with SGRT (from 5.4 to 6.3 min), but this did not impact their workflow.

### 4.2. Head and Neck/Brain

SGRT technique for H and N and brain CA patients, an open-face mask is necessary to immobilize the patient’s head so that the patient’s surface can be tracked. Open-face masks have been found to have comparable results in terms of intrafractional motion to closed masks [43] while providing lower levels of anxiety and claustrophobia [43,44].

SGRT positioning in H and N and brain regions can reduce residual errors compared with laser/tattoo positioning. These results can be explained by a good correlation between the patient’s surface and the internal tumor [20]. Wei et al. [30] mentioned that the changes in the patient anatomy, such as tumor response and weight loss during radiation therapy, could not be accurately captured by markers. By contrast, with the recording of a reference image for each treatment session with SGRT after alignment with IGRT imaging serving as a baseline for the next day’s treatment, these changes could be detected. Chen et al. [31] mentioned that lower median residual errors were observed in the SGRT group, in which a customized mouth bite in the open-face mask was used, in comparison with the closed-mask group. The mouth bite helped improve the interfractional position repeatability of the patients’ mandibles. Additionally, the implementation of SGRT allowed for the adjustment of the patient’s head to the reference surface before wearing the open-face mask, thereby reducing errors before the CBCT scan.

On the other hand, Wei et al. [30] discovered that SGRT increased rotational errors, particularly in pitch. This could be explained by the fact that the H and N anatomical region is not entirely rigid owing to its flexible bony structures [45]. As a result, internal rotations may occur, which differ from the reference positions. Therefore, the rotational changes may not be precisely reflected by the anterior skin surface used for registration in SGRT. It is noteworthy to mention that Wei et al. [30] analyzed the H and N region stratified in three treatment sites because of the varying anatomical levels and depths of these sites. A study conducted by Lee et al. [46] discovered a noticeable rise in the mean translational setup errors in patients with nasopharynx and brain CA as the vertical distance between the skin and the isocenter increased. This significant increase indicated that the ROI location in the SGRT setup was biased toward the skin-to-isocenter distance (*p* < 0.001). This supports the hypothesis that there is a mild dependence on the distance between the skin and the isocenter. 

Another parameter that could affect the accuracy of positioning with SGRT in patients with H and N and brain CA is changes in patients’ facial expressions. Bry et al. [47] conducted a study to determine how expressions affect the accuracy of SGRT while wearing an open-face mask. The average deviation of the ROI was 0.8 ± 0.9 mm when the patient’s eyes were open, which was significantly higher than the deviation of 0.3 ± 0.3 mm with the eyes closed. Additionally, the average displacement was 1.3 ± 1.3 mm for larger ROIs and 2.0 ± 1.8 mm for smaller ROIs, resulting from fear and annoyance. Facial movements can result in incorrect information about the body surface during SGRT treatment. Therefore, they suggested that patients positioned with SGRT should be instructed to keep their eyes closed throughout the treatment, the ROI size should be as large as possible, and the area around the eye should be excluded.

Wei et al. [30] found that when SGRT guidance was used, the average setup time for all three treatment sites was shorter. However, no significant difference was observed. The most considerable reduction in time between the SGRT and non-SGRT groups was noticed in the nasopharynx/sinonasal group, with 6.14 ± 2.44 min versus 4.18 ± 2.16 min (*p* = 0.09). E. Flores-Martinez et al. [29] found that the setup time for patients treated before the implementation of SGRT was 283 ± 84 s, and with SGRT, the mean time was 293 ± 89 s. However, the imaging time was reduced from 122 ± 95 s to 89 ± 46 s using SGRT. Therefore, the total treatment time was reduced.

### 4.3. Thorax

Various studies have revealed that the two positioning methods for patients with CA in the thorax region yield comparable results. Walter et al. [19] found non-statistically significant differences between the two techniques. This could be attributed to the fact that fractions in the thorax region had a small sample size. Qubala et al. [33] suggested that the smaller translational errors observed in the patients with laser/tattoo setup could be due to the use of free-breathing CT scans in thorax treatment planning, which are influenced by the interplay between scanning and free breathing. As a result, the patient’s current position can deviate from the reference position, particularly in vertical and longitudinal errors.

In a study conducted by Blake et al. [32] during the COVID-19 pandemic in 2020, the SGRT protocol was used for patient positioning. The researchers concluded that SGRT could be utilized as a technique to reduce close contact between patients and staff, providing increased safety without compromising established standards for setup accuracy, as confirmed by kVCBCT imaging.

Concerning the setup time in the thoracic region, Qubala et al. [33] discovered that SGRT resulted in a statistically significant reduction of 15% in patient setup time.

### 4.4. Abdomen 

In two of the three studies involving patients with abdominal CA, the residual errors observed with SGRT were similar or worse than those of conventional positioning techniques.

In the abdominal region, the sample size of fractions studied by Walter et al. [19] was small, which could have affected the results. Furthermore, the SGRT system might deviate in the abdominal region due to the poor correlation between the external patient’s surface and internal organ motion [48].

To ensure the accuracy of the SGRT technique in the abdominal area, it is crucial to choose the appropriate ROI carefully. Defining the ROI requires thorough investigation, as the clinical goal is to reproduce the body and diaphragm position, resulting in a better representation of the position of the liver and pancreas during treatment. In their study, Song et al. [49] analyzed six ROIs for the DIBH SGRT setup in the abdominal region. These included two rectangular ROIs, two ribcage ROIs on either side, and two triangular ROIs for soft tissue at the center. They found that the rectangular ROI that covered the central abdominal area and the lateral region with minimal rib support had the strongest correlation (Pearson coefficient = 0.67) with the diaphragm dome. They also recommended selecting an ROI for free breathing that remains stable with minimal motion or deformation, with a surface including the ribs being the optimal choice.

### 4.5. Pelvis

Mannerberg et al. [34] and Qubala et al. [33] showed that SGRT provided improved results in patient positioning compared with lasers/tattoos in the pelvic area. The statistically significant differences that were calculated favored SGRT. The improvements could be explained by the fact that the SGRT setup uses the entire topography of the patient, while three-point localization only uses a single tattoo on each patient’s side [34]. Furthermore, as Qubala et al. [33] mentioned, SGRT provides a more consistent workflow for every patient compared with positioning with a certain number of skin marks. Thus, it is less reliant on the individual skills of personnel.

On the other hand, there are some limitations when SGRT is used for patient positioning in the pelvic region. The hair on the patient’s surface in the treatment area compromises patient positioning accuracy due to the loss of surface calculation points [19]. Moreover, the most critical factor is the day-by-day variations in the bladder and bowel filling and interfraction movement of the target (e.g., the prostate), which are not evident on the patient’s surface [50].

Reducing the time in the pelvic region is crucial for patients with conditions like prostate cancer. This is because it minimizes uncertainties associated with the intrafractional motion of the prostate. According to Mannerberg et al. [34], the median setup time using SGRT was 2:50 min (range 1:32–6:56 min), while the setup time using tattoos took 3:28 min (range: 1:42–12:57 min). The difference was statistically significant (*p* < 0.001). Similarly, research by Qubala et al. [33] showed that using SGRT reduced patient setup time for pelvic treatments by 18%. In fact, 53% of the patients in their study had faster positioning times with SGRT, and 87.5% had shorter matching times than with tattoos.

### 4.6. SGRT and Intrafractional Monitoring

Covington et al. [51] studied patients who received SRS/SRT and were monitored for intrafractional motion by an SGRT system. They found that during gantry motion when camera pods are blocked, the median magnitude was below 1 mm. At 270°, the couch angle was found to have a more significant deviation in the longitudinal direction (median value: −0.64 mm) than the value observed at the zero-couch angle. Also, interestingly, they found that in 32 fractions, SGRT detected that the intrafractional motion exceeded the tolerance of 1 mm in the translational directions, and 19 fractions (60%) were found to be in agreement with kVCBCT. Therefore, they recommended that SGRT is a feasible option for monitoring intrafractional motion during SRS.

Gregucci et al. [52] assessed the intrafraction SGRT data compared to post-treatment kVCBCT findings in patients with intracranial diseases. The cross-check between the two modalities validated the deltas calculated by the SGRT system. Hence, they concluded that SGRT can be utilized for intrafractional monitoring, omitting post-treatment kVCBCT. 

Heinzerling et al. [53] found no statistically significant difference between the detected motion by SGRT and the resulting kVCBCT shifts in the translational vector (*p* = 0.676) in patients who were treated with SBRT for lung CA. The magnitudes of detected motion and resulting internal shifts in all six directions were observed to be numerically identical. Of the 34 fractions in which SGRT detected potential intrafractional motion of 2 mm or greater, repeated kVCBCT detected internal target motion of at least 2 mm in 25 (73.5%) of those fractions. They suggested that SGRT can detect patient motion, which may be a surrogate for detecting target displacement outside of PTV margin expansion for SBRT treatment in the lungs.

Nguyen et al. [54] studied thirteen lung CA SBRT patients treated on O-ring linacs using the SGRT-based DIBH technique with a gating window of ±1 mm. In evaluating the intrafractional motion that SGRT tracked, three control kVCBCTs were performed: one before the first treatment delivery, one halfway through the treatment, and one before the last VMAT arc. The real-time values of SGRT correlated with the kVCBCT shifts in the longitudinal (*p <* 0.001), lateral (*p* = 0.001), and roll (*p* = 0.05) directions. No correlation was found for the vertical, yaw, or pitch directions. Bland and Altman plots proved that the mean difference between the SGRT and CBCT shifts remained <0.03 mm in the vertical direction and <0.4 mm/° in the other five translational and rotational directions, indicating that the tumor remained within the PTV whenever the surface position was within SGRT tolerance. Finally, they concluded that ring-mounted SGRT systems with tight thresholds are sufficient to monitor intrafractional tumor motion without additional control kVCBCT.

A study conducted by Zeng et al. [55] included 14 patients with gastrointestinal cancer who underwent SGRT-based DIBH with a gating window of ±3 mm. The SGRT system real-time monitoring was evaluated with kV image acquisition triggered every 20° or 40° of gantry rotation during treatment delivery. The registration of the kV image with the DRRs was performed retrospectively according to at least one radiopaque marker, which was implanted in every patient near or within the target. They found that although the SGRT ROI motion was within the gating window, the internal movement amplitude was often greater, about 10 mm on average and 20 mm in extreme cases. When they compared the results of this study with those of their previous one [56] with the real-time position management system (RPM, Varian Medical Systems, Palo Alto, CA, USA), they found that in the RPM cohort, a >5 mm displacement was observed in 10% of the images analyzed compared with 19% in the current SGRT cohort. Four patients (27%) in the SGRT cohort exhibited at least a 3 mm displacement compared with 12% in the RPM cohort, and one case exhibited 5 mm (7%) compared with none in the RPM cohort. However, the gating window in the RPM study was ±1.5 mm, which was smaller than that in the SGRT study (±3 mm). This was less restrictive and may have contributed to the more significant residual motion. Furthermore, the extended arms of the kV source and imager may have also affected the accuracy of the SGRT system by blocking the system’s cameras during the gantry rotation of the arc treatments. One more limitation of their study was that the shape of the ROI that they used may have needed to be more optimal. They concluded that using SGRT as a solitary technique for real-time motion monitoring is discouraged for this kind of treatment. However, many factors could have affected their results. 

One of the most significant advantages of SGRT is the ability to monitor patients’ intrafractional motion without additional dosage. Real-time monitoring becomes essential, especially in stereotactic applications, such as stereotactic radiosurgery (SRS), stereotactic radiotherapy (SRT), and stereotactic body radiotherapy (SBRT). When an SGRT system is installed, the system must be calibrated with respect to the treatment and the kV imager isocenters. Quality assurance (QA) is vital to define how different parameters affect the accuracy of the system. Examples include the effectiveness of the system in non-zero couch angles as well as when there is occlusion of one or two SGRT units from linac components, ROI shape/position/size, and the depth of the isocenter in regard to the patient’s surface [57]. Recently, Task Group 302 (TG-302) was published. It provides commissioning and ongoing QA requirements for SGRT as part of a comprehensive QA program that includes risk assessment [58]. 

### 4.7. SGRT and Imaging Dose Reduction 

SGRT is a state-of-the-art technique that optimizes patient positioning and can lead to dose reduction. However, as discussed in the previous sections, some parameters can impact the technique’s accuracy. Hence, SGRT is not a surrogate for IGRT but is complementary to it. Therefore, from this point of view, SGRT cannot lead to dose reduction. Nevertheless, using SGRT can reduce the patient’s dose in other ways. 

As discussed previously, SGRT is a feasible technique for tracking intrafractional motion, especially in the head and thorax treatment areas. In these cases, the IGRT modality, which is used for intrafractional monitoring either during or at the end of treatment, could be replaced by SGRT, leading to lower doses for the patients [51,52].

Furthermore, it has been proven by several authors that in workflows involving 2D kV imaging for patient positioning before 3D kV imaging verification (e.g., kVCBCT), an SGRT module can take the place of the 2D kV imaging step. This results in reduced imaging doses for patients while maintaining the accuracy of the positioning [42,53,59].

According to the AAPM TG-180 [60], the typical doses of IGRT procedures are as follows: 1–5 cGy organ doses for a typical pair of orthogonal 6 MV portal images, 0.6–1.2 cGy/MU organ doses for MVCBCT, 1–9 cGy to soft tissues and 6–29 cGy to bones for a single kVCBCT procedure, and on the order of 0.1 cGy for fixed double X-ray tube systems that use projected images for patient localization. This highlights the fact that the optimization of imaging procedures in radiotherapy is of high importance. The implementation of SGRT can reduce residual errors, as they are determined by IGRT. Specifically, Flores-Martinez et al. [29] found that without SGRT, the need for repositioning and reimaging was necessary for 11.1% of the fractions for H and N cases. Once SGRT was incorporated into the workflow, the percentage of fractions requiring repositioning was reduced to 5.5%. Qubala et al. [33] observed that six pelvic cases and five thoracic cases positioned with tattoos needed reimaging, while only one case of each anatomical site needed reimaging with SGRT positioning. Blake et al. [32] discovered that the number of times that complete re-setup and reimaging were needed was reduced from 3% with tattoos to only 0.3% when using SGRT. The aforementioned studies show that SGRT can reduce the need for patient repositioning/reimaging.

### 4.8. Future Perspectives

SGRT holds profound implications for advancing the precision and efficacy of radiation therapy treatments. As technology evolves, SGRT is poised to emerge as a pivotal tool in the optimization of treatment delivery and patient care. The integration of SGRT with advanced imaging modalities, such as CBCT, promises a paradigm shift in treatment accuracy. This amalgamation enables the synchronization of real-time external surface data with high-resolution internal anatomical information, facilitating an unprecedented level of patient-specific positioning. Furthermore, the incorporation of artificial intelligence (AI) augments SGRT’s potential by providing predictive modeling and adaptive capabilities. AI algorithms can analyze historical patient data to anticipate real-time motion patterns, enabling preemptive patient positioning adjustments during treatment sessions. This predictive prowess has the potential to mitigate treatment errors arising from patient motion, ensuring precise dose delivery to intended targets.

The markerless and non-invasive nature of SGRT, coupled with AI-driven adaptations, enhances patient comfort and safety. This transformative approach also holds the promise of expediting treatment workflows. Reduced repositioning requirements could potentially result in shorter treatment times, alleviating the physical and psychological burden on patients.

Moreover, the evolving landscape of SGRT is intertwined with the expansion of patient-centered and personalized treatment regimens. The convergence of SGRT with AI, imaging modalities, and adaptive strategies underscores a trajectory toward patient-specific treatments that account for dynamic anatomical changes and intrinsic biological responses. However, this transformative path demands continued research, rigorous validation studies, and seamless integration with existing clinical protocols.

The future of SGRT will be characterized by a synergy of technological advancements encompassing advanced imaging, AI integration, and patient-centered care. The potential to enhance treatment precision, reduce errors, and improve patient experience positions SGRT as a cornerstone of future radiation therapy practices. By embracing this trajectory, the field stands to realize a new era of treatment accuracy, efficiency, and patient-centered care.

## 5. Conclusions

According to the available literature, SGRT is a highly effective positioning technique that can be used across all treatment sites and for all types of cancer. It has been demonstrated to produce results that are at least as good as those achieved using the conventional methods of tattoos/lasers. The transition from the era of three-point markers to the new era of thousands of points can significantly reduce the uncertainties in patient positioning, treatment time, and the post-treatment psychological effects of tattoos. Moreover, by implementing SGRT, the imaging dose to the patient can be minimized by eliminating the need for imaging with the IGRT modality for intrafractional monitoring, 2D kV imaging for patient positioning before 3D kV imaging verification, and repositioning/reimaging. SGRT can also be utilized as a reliable technique for monitoring intrafractional motion, increasing treatment accuracy and precision while providing a safe and comfortable environment for the patient. 

## Data Availability

No data.
